# Prognostic value of the systemic immune-inflammation index in patients with upper tract urothelial carcinoma after radical nephroureterectomy

**DOI:** 10.1186/s12957-023-03225-0

**Published:** 2023-10-26

**Authors:** Zhenkai Luo, Yangxuanyu Yan, Binbin Jiao, Tao Huang, Yuhao Liu, Haijie Chen, Yunfan Guan, Zhenshan Ding, Guan Zhang

**Affiliations:** 1 Graduate School of Peking Union Medical College and Chinese Academy of Medical Sciences, No. 17 Nanli, Panjiayuan, Chaoyang District, Beijing, 100730 China; 2 Department of Colorectal Surgery, National Cancer Center/National Clinical Research Center for Cancer/Cancer Hospital, Chinese Academy of Medical Sciences and Peking Union Medical College, No. 17 Nanli, Panjiayuan, Chaoyang District, Beijing, 100021 China; 3 Department of Urology, China-Japan Friendship Hospital, Yinghuadong Road, Chaoyang District, Beijing, 100029 China; 4 Peking University China-Japan Friendship School of Clinical Medicine, Yinghuadong Road, Chaoyang District, Beijing, 100029 China; 5 Department of Urology, Beijing Chao-Yang Hospital, Capital Medical University, Gongren Tiyuchang Nanlu, Chaoyang District, Beijing, 100020 China; 6 Department of Urology, The First Affiliated Hospital of Nanjing Medical University, No.300. Guangzhou Road, Nanjing, 210029 China

**Keywords:** Systemic immune-inflammation index, Upper tract urothelial carcinoma, Extraurothelial recurrence, Radical nephroureterectomy, Risk factor

## Abstract

**Background:**

To investigate the prognostic significance of the systemic immune-inflammation index (SII) for patients with upper tract urothelial carcinoma (UTUC) after radical nephroureterectomy (RNU) and develop nomogram models for predicting overall survival (OS), intravesical recurrence (IVR), and extra-urothelial recurrence (EUR).

**Methods:**

We retrospectively studied the clinical and pathological features of 195 patients who underwent RNU for UTUC. All patients were randomly divided into a training cohort (99 cases) and a validation cohort (96 cases). The training cohort was used to develop nomogram models, and the models were validated by the validation cohort. The least absolute shrinkage and selection operator (LASSO) regression and Cox regression were performed to identify independent predictors. The concordance index (C-index), receiver operator characteristics (ROC) analysis, and calibration plot were used to evaluate the reliability of the models. The clinical utility compared with the pathological T stage was assessed using the net reclassification index (NRI), integrated discrimination improvement (IDI), and decision curve analysis (DCA).

**Results:**

SII was an independent risk factor in predicting OS and EUR. The C-index values of the nomogram predicting OS, IVR, and EUR were 0.675, 0.702, and 0.756 in the training cohort and 0.715, 0.756, and 0.713 in the validation cohort. A high level of SII was correlated with the invasion of the mucosa, muscle layer of the ureter, nerves, vessels, and fat tissues.

**Conclusion:**

We developed nomogram models to predict the OS, IVR, and EUR of UTUC patients. The efficacy of these models was substantiated through internal validation, demonstrating favorable discrimination, calibration, and clinical utility. A high level of SII was associated with both worse OS and shorter EUR-free survival.

**Supplementary Information:**

The online version contains supplementary material available at 10.1186/s12957-023-03225-0.

## Background

Upper tract urothelial carcinoma (UTUC) accounts for 5–10% of urothelial carcinomas [1]. Though radical nephroureterectomy (RNU) with bladder cuff removal is the standard treatment of UTUC patients, the tumors were found to be invasive at diagnosis in 60% of cases [2]. The disease recurrence in the bladder or non-bladder sites is frequent [3]. Many studies have focused on the pre-, intra-, and postoperative prognostic factors of patients with UTUC after RNU [4–7]. According to the European Association of Urology (EAU) Guidelines on UTUC, template lymphadenectomy, and perioperative platinum-based combination chemotherapy should be considered in patients with high-risk tumors [2]. Enhancing comprehension of prognostic factors and constructing a predictive model can facilitate the identification of patients at high risk of recurrence, thereby necessitating the implementation of more rigorous therapeutic and monitoring interventions.

Preoperative prognostic factors encompass various variables such as patient age, tobacco usage, tumor focality, tumor location, grade, hydronephrosis, and inflammation-related indicators, among others [6]. However, the accuracy of tumor pathological features obtained through uroscopy is limited [8]. Furthermore, preoperative ureteroscopy has been identified as a risk factor for intravesical recurrence (IVR) and has a negative impact on the prognosis of patients with UTUC after RNU [9, 10]. Additionally, imaging modalities such as computed tomography (CT) and magnetic resonance imaging (MRI) pose challenges in detecting microscopic invasion and are inadequate for determining personalized treatment approaches [6, 11, 12].

Inflammation plays a contributing role in the initiation and advancement of various cancers [13]. Numerous inflammation- and immune-related factors have been identified as having prognostic value for oncological outcomes in patients with UTUC following RNU [7, 14], including the neutrophil-to-lymphocyte ratio (NLR), platelet-to-lymphocyte ratio (PLR), and lymphocyte-to-monocyte ratio (LMR). Increased NLR, PLR, and LMR have been linked to a heightened risk of recurrence and poorer survival rates [15]. The systemic immune-inflammation index (SII), which is an integrated immune and inflammatory index derived from peripheral lymphocyte, neutrophil, and platelet counts, has been identified as an independent prognostic indicator in various cancer types such as gastric cancer, colorectal cancer, hepatocellular cancer, and lung cancer [16–19]. A meta-analysis has demonstrated that a higher SII value is significantly associated with poorer survival outcomes in urological cancers, including prostate cancer and urothelial carcinoma [20].

The predictive efficacy of SII in patients with UTUC after RNU has been assessed in several studies. These studies have reported that a high SII is an independent predictor of poorer recurrence-free survival (RFS), cancer-specific survival (CSS), and overall survival (OS) [15, 21]. Additionally, an elevated SII is associated with an increased risk of muscle-invasive and non-organ-confined disease following RNU [15, 21]. Moreover, the SII has been shown to be a significant prognostic factor for bladder recurrence [11]. However, the prognostic significance of SII in relation to extra-urothelial recurrence (EUR) remains unexplored, and the potential correlation between tumor status and SII has not been thoroughly examined. This study aims to assess the predictive value of SII for survival outcomes and recurrence in patients with UTUC, investigate the association between tumor status and SII, and construct a predictive model based on significant prognostic factors.

## Methods

### Patient selection

This retrospective study was approved by the Institutional Research Ethics Committee of China-Japan Friendship (2021–40-K24). Informed consent was obtained from all eligible participants in advance. We retrospectively collected the information of patients diagnosed with UTUC who received RNU treatment at our hospital from 2009 to 2020, and all patients’ details have been de-identified. We included the patients who meet the following criteria: (1) patients with UTUC confirmed pathologically, (2) patients with primary disease, (3) patients with unilateral onset, and (4) patients subject to RNU combined with cystic sleeve resection. Patients were excluded according to the following criteria: (1) patients with bilateral UTUC, (2) patients subject to no RNU combined with cystectomy, and (3) patients with metastatic uroepithelial carcinoma.

### Follow-up and cohort definition

We monitored patients every 3 months during the first year after surgery, every 6 months through the third year, and once a year thereafter. Follow-up data included blood tests, cystoscopic examination, urinary system ultrasound, chest and abdomen CT, urine exfoliated cytology, and urography. Selective bone scan, PET/CT, or MRI were performed if clinically indicated. OS was defined as the time from the date of RNU to death from any cause. Intravesical recurrence-free survival (IVRFS) was defined as the time from the date of RNU to the date of the first IVR according to cystoscopic examination. Extraurothelial recurrence-free survival (EURFS) was defined as the time from the date of RNU to the date of the first EUR according to imaging examination. The patients were randomly divided into the training and validation cohorts with a ratio of 1:1 using the R function “createDataPartition.” The training set was utilized for the development of nomograms, determination of the cutoff value for SII, and serum aspartate transaminase/alanine transaminase (ALT/AST), as well as risk stratification. The findings derived from the training set were subsequently validated in the validation cohort.

### Data collection

Sixteen variables were included: age, sex, history of hypertension, history of diabetes mellitus (DM), body mass index (BMI), tumor side, tumor location, tumor grade, pathological tumor stage, tumor size, SII, ALT/AST ratio, estimated glomerular filtration rate (eGFR), urine cytology, ureteroscopy, and presence of hydronephrosis. Pretreatment SII values were assessed within 30 days prior to RNU. SII was calculated as platelet count × neutrophil/lymphocyte count. The optimal SII cutoff value was defined by creating a time-dependent receiver operating characteristic (ROC) curve with OS as the endpoint to yield the highest Youden index value. The overall study population was divided into two separate SII groups (> 470 vs. ≤ 470) according to the optimal cutoff. The preoperative eGFR was calculated using the following formula: 186 (serum creatinine)^(–1.154)*(age)^(–0.203)*(0.742 if female). Patients with an eGFR lower than 60 ml/min/1.73 m^2^ were considered to have chronic kidney disease. Tumor stages were defined pathologically based on the American Joint Committee on Cancer (AJCC) Tumor, Node, Metastasis (TNM) classification (eighth edition). Tumor grades were defined using the 2008 World Health Organization (WHO) classification. The tumor location is marked according to the location of the dominant tumor. Positive urine cytology was defined as the presence of tumor cells or abnormal cells in preoperative samples. Conversely, negative urine cytology was defined as an evaluation that yielded negative results. The evaluation of all histopathological slides was conducted by the senior pathologist.

### Statistical analysis

Predictive models were constructed through the utilization of Cox regression with the least absolute shrinkage and selection operator (LASSO) regression. In order to optimize parameter selection within the LASSO regression, a tenfold cross-validation was conducted. Subsequently, a multivariable Cox regression analysis was employed to ascertain independent risk factors, which were then integrated into the nomograms. Additionally, collinearity testing was conducted using the variance inflation factor (VIF), whereby a VIF value exceeding 4.0 was deemed indicative of multicollinearity. Variables exhibiting a VIF value surpassing 4.0 were consequently excluded from the model. The 1-/3-/5-year OS, IVRFS, and EURFS probabilities were estimated using the nomograms. The discriminations of the models were evaluated using concordance indexes (C-index) calculated by bootstrapping and time-dependent area under curve (AUC). Calibration curves were calculated to assess the predictive ability. We set time-dependent ROC curves with OS, IVRFS, and EVRFS as the endpoint, respectively, to define the optimal cutoff point for risk stratifications.

We expressed the categorical variables as the frequency (percentage). Some results were shown as interquartile ranges (IQRs). All variables were categorized using the cutoff set from time-dependent ROC or previous reports. The association of variables was assessed with the *χ* ^2^ test and Fisher’s exact test. The net reclassification index (NRI), integrated discrimination improvement (IDI), and decision curve analysis (DCA) were used for the evaluation of prediction improvement compared with prediction based on pathological tumor staging alone. All *P* values were two-tailed, and *P* < 0.05 was considered statistically significant. R software (Version 4.2.2) and IBM SPSS Statistics (Version 24) were utilized to complete all statistical analyses and figures.

## Results

### Characteristics of patients and disease

A cohort of 195 patients who met the specified inclusion and exclusion criteria were included in the study. Data on 16 pre- or peri-operative variables, tumor invasion, and the duration of overall survival, intravenous revascularization, and endovascular ureteral reimplantation were collected. The patients were randomly divided into training and validation groups in a 1:1 ratio. The median follow-up period was 43 months, with an IQR of 26.5–70.5 months. The clinical characteristics of all patients were summarized in Table 1. There were no significant differences observed in pre- and peri-operative and demographic parameters among the patients.

**Table 1:** Clinical and pathological characteristics of patients

Variable	Training cohort *N* = 99	Validation cohort *N* = 96	*P* value
Gender			
Female	55 (55.6%)	56 (58.3%)	0.805
Male	44 (44.4%)	40 (41.7%)	
Age			
> 65	60 (60.6%)	55 (57.3%)	0.745
≤ 65	39 (39.4%)	41 (42.7%)	
Hypertension history			
No	53 (53.5%)	47 (49.0%)	0.620
Yes	46 (46.5%)	49 (51.0%)	
Diabetes history			
No	78 (78.8%)	80 (83.3%)	0.531
Yes	21 (21.2%)	16 (16.7%)	
BMI			
< 24	43 (43.4%)	45 (46.9%)	0.735
≥ 24	56 (56.6%)	51 (53.1%)	
Tumor side			
Left	51 (51.5%)	51 (53.1%)	0.935
Right	48 (48.5%)	45 (46.9%)	
Location			
Both	24 (24.2%)	16 (16.7%)	0.174
Renal pelvis	38 (38.4%)	32 (33.3%)	
Ureter	37 (37.4%)	48 (50.0%)	
Tumor size			
> 3	31 (31.3%)	40 (41.7%)	0.176
≤ 3	68 (68.7%)	56 (58.3%)	
Tumor stage			
≤ 2	71 (71.7%)	75 (78.1%)	0.386
≥ 3	28 (28.3%)	21 (21.9%)	
Neural or vascular invasion			
No	81 (81.8%)	85 (88.5%)	0.264
Yes	18 (18.2%)	11 (11.5%)	
Renal sinus invasion			
No	84 (84.8%)	90 (93.8%)	0.076
Yes	15 (15.2%)	6 (6.25%)	
Pararenal invasion			
No	81 (81.8%)	88 (91.7%)	0.070
Yes	18 (18.2%)	8 (8.33%)	
Mucosa invasion			
No	78 (78.8%)	81 (84.4%)	0.412
Yes	21 (21.2%)	15 (15.6%)	
Subepithelial invasion			
No	93 (93.9%)	94 (97.9%)	0.279
Yes	6 (6.06%)	2 (2.08%)	
Muscle invasion			
No	53 (53.5%)	56 (58.3%)	0.596
Yes	46 (46.5%)	40 (41.7%)	
Fat invasion			
No	84 (84.8%)	82 (85.4%)	1.000
Yes	15 (15.2%)	14 (14.6%)	
Cancer embolus			
No	86 (86.9%)	84 (87.5%)	1.000
Yes	13 (13.1%)	12 (12.5%)	
Ureteral ends invasion			
No	94 (94.9%)	94 (97.9%)	0.445
Yes	5 (5.05%)	2 (2.08%)	
Lymph node invasion			
No	95 (96.0%)	93 (96.9%)	1.000
Yes	4 (4.04%)	3 (3.12%)	
Hydronephrosis			
No	71 (71.7%)	53 (55.2%)	0.025
Yes	28 (28.3%)	43 (44.8%)	
Urine cytology			
Abnormal	59 (59.6%)	53 (55.2%)	0.635
Normal	40 (40.4%)	43 (44.8%)	
Ureteroscopy			
No	39 (39.4%)	43 (44.8%)	0.536
Yes	60 (60.6%)	53 (55.2%)	
SII			
> 470	51 (51.5%)	41 (42.7%)	0.277
≤ 470	48 (48.5%)	55 (57.3%)	
ALT/AST			
> 0.55	72 (72.7%)	65 (67.7%)	0.542
≤ 0.55	27 (27.3%)	31 (32.3%)	
eGFR			
< 60	67 (67.7%)	63 (65.6%)	0.879
≥ 60	32 (32.3%)	33 (34.4%)	
Tumor stage			
≤ 2	71 (71.7%)	75 (78.1%)	0.386
≥ 3	28 (28.3%)	21 (21.9%)	
Tumor size			
> 3	31 (31.3%)	40 (41.7%)	0.176
≤ 3	68 (68.7%)	56 (58.3%)	
Tumor grade			
High	92 (92.9%)	83 (86.5%)	0.210
Low	7 (7.07%)	13 (13.5%)	

*BMI* Body mass index, *SII* Systemic immune-inflammation index, *ALT/AST* Serum aspartate transaminase/alanine transaminase, *eGFR* Estimated glomerular filtration rate

### Variable screening

We first utilized LASSO Cox regression to screen possible prognostic factors (Figure S1). When the minimum lambda was 0.022, 0.034, and 0.043, 18, 12, and 8 potential predictors for OS, IVR, and EUR were screened out in the training cohort, respectively. Then, we established a multivariate Cox model to identify independent risk factors significantly affecting OS, IVRFS, or EURFS in patients with UTUC after RNU. Finally, we identified several independent risk factors for predicting OS, including BMI, gender, SII, and tumor stage (Table 2). Additionally, urine cytology, eGFR, and tumor stage were found to be independent risk factors for predicting IVR (Table S1), while BMI, gender, SII, hydronephrosis, and tumor stage were independent risk factors for EUR (Table S2).

**Table 2:** Univariate and multivariate analyses of predictive factors for overall survival

Variable	*P* value	HR (95% CI for HR)	*P* value	HR (95% CI for HR)
Age	0.63	0.84 (0.43–1.7)	0.41	0.7 (0.3–1.6)
BMI	0.33	1.4 (0.7–2.8)	0.03	2.9 (1.1–7.8)
Urine cytology	0.41	1.3 (0.68–2.6)	0.26	1.5 (0.72–3.3)
Diabetes history	0.58	0.76 (0.29–2)		
eGFR	0.6	0.83 (0.4–1.7)	0.25	0.62 (0.28–1.4)
Gender	0.035	2.1 (1.1–4.1)	0.013	2.8 (1.2–6.4)
Tumor grade	0.66	0.72 (0.17–3)	0.22	2.7 (0.55–13)
Hypertension history	0.54	0.81 (0.41–1.6)	0.14	0.54 (0.23–1.2)
ALT/AST	0.77	1.1 (0.51–2.5)		
Hydronephrosis	0.2	1.6 (0.78–3.2)	0.1	2.3 (0.84–6)
Renal pelvic carcinoma	0.073	0.5 (0.23–1.1)	0.1	0.44 (0.16–1.2)
Ureteral carcinoma	0.33	1.4 (0.71–2.7)		
Tumor in both	0.32	1.4 (0.7–3)	0.068	2.5 (0.93–6.9)
Tumor side	0.62	0.84 (0.43–1.6)	0.74	0.87 (0.4–1.9)
SII	0.08	0.54 (0.27–1.1)	0.0075	0.32 (0.14–0.74)
Tumor size	0.55	0.81 (0.4–1.6)	0.21	0.61 (0.28–1.3)
Tumor stage	0.07	1.9 (0.95–3.8)	0.004	3.4 (1.5–7.8)
Ureteroscopy	0.53	1.2 (0.62–2.5)		

*BMI* Body mass index, *SII* Systemic immune-inflammation index, *ALT/AST* Serum aspartate transaminase/alanine transaminase, *eGFR* Estimated glomerular filtration rate

### Nomogram construction and validation

**Fig. 1:**
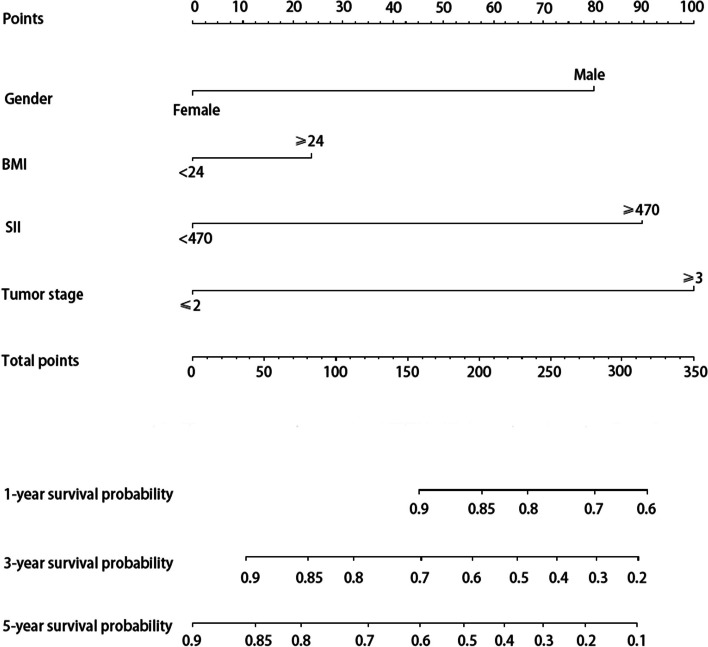
Nomograms for a 1-, 3-, and 5-year OS prediction of patients with UTUC after RNU

**Fig. 2:**
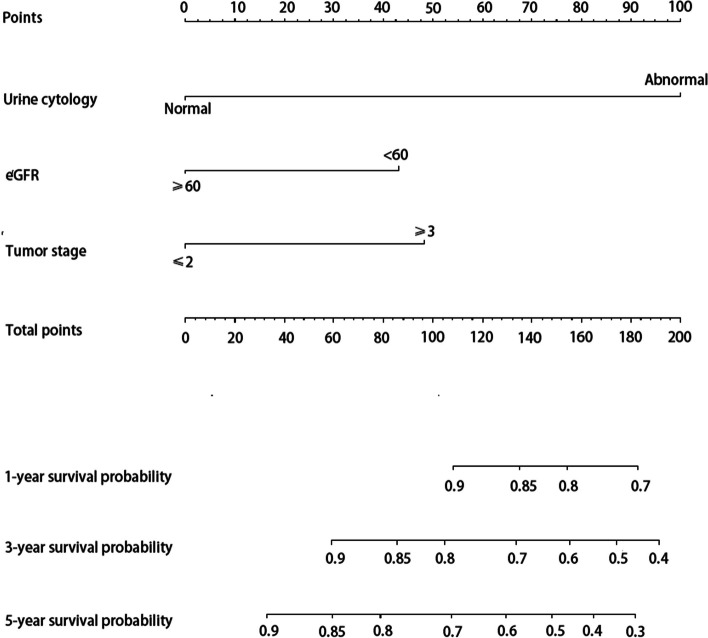
Nomogram for a 1-, 3-, and 5-year IVRFS prediction of patients with UTUC after RNU

**Fig. 3:**
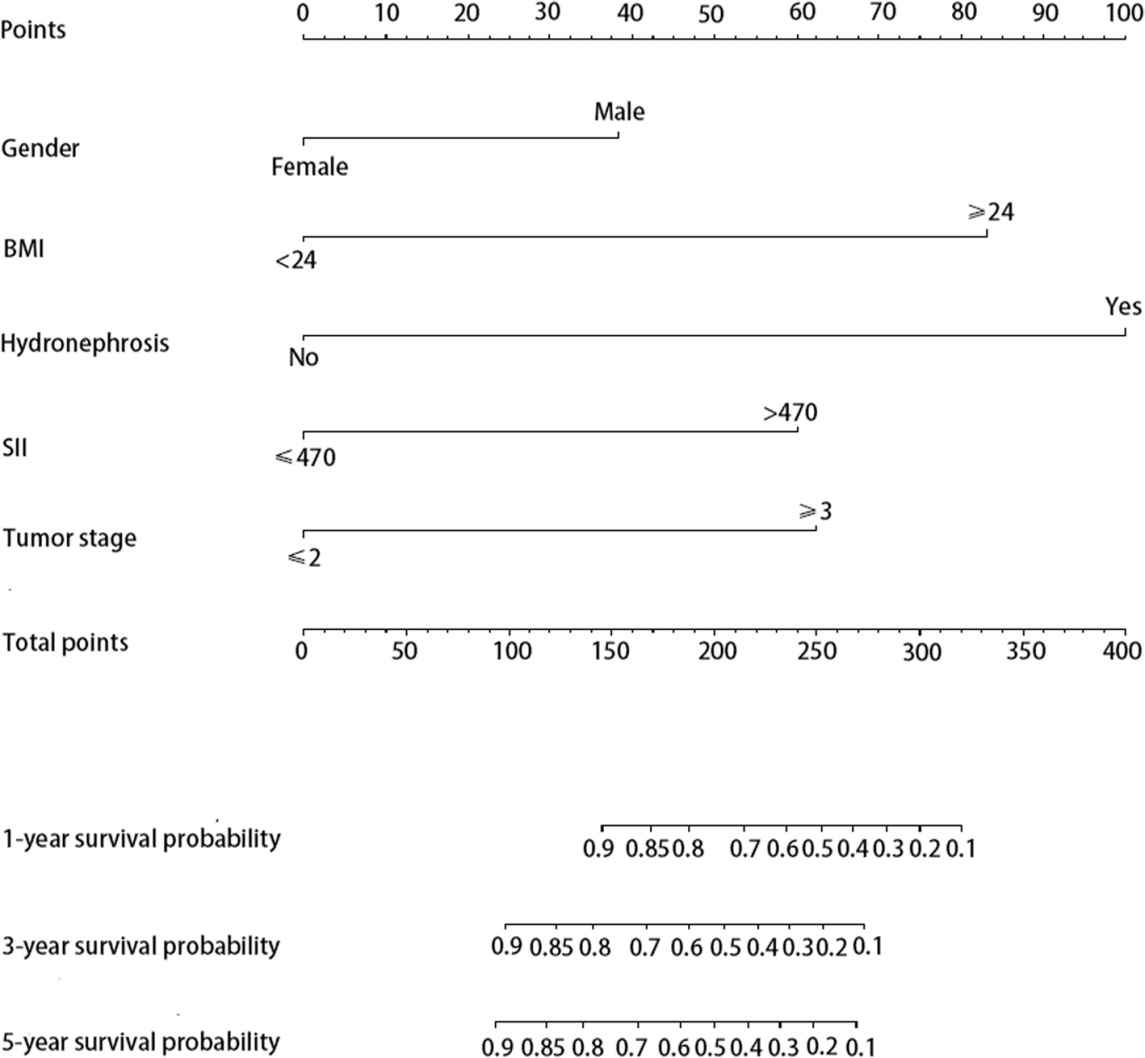
Nomogram for a 1-, 3-, and 5-year EURFS prediction of patients with UTUC after RNU

**Fig. 4:**
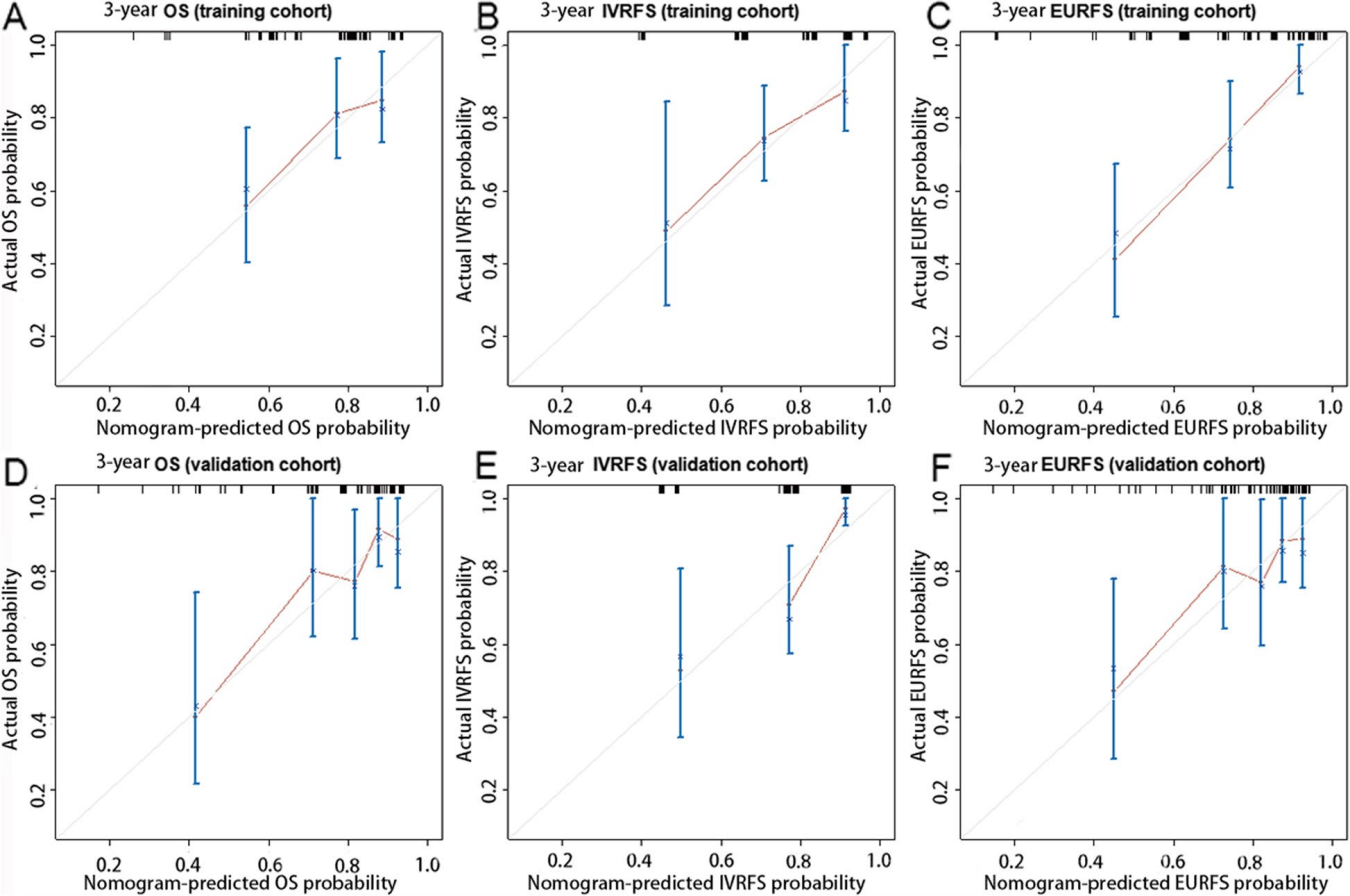
The calibration curves of the OS nomogram in the training cohort (**A**) and at the validation cohort (**D**). The calibration curves of the IVRFS nomogram in the training cohort (**B**) and at validation cohort (**E**). The calibration curves of the EURFS nomogram in the training cohort (**C**) and at the validation cohort (**F**)

**Fig. 5:**
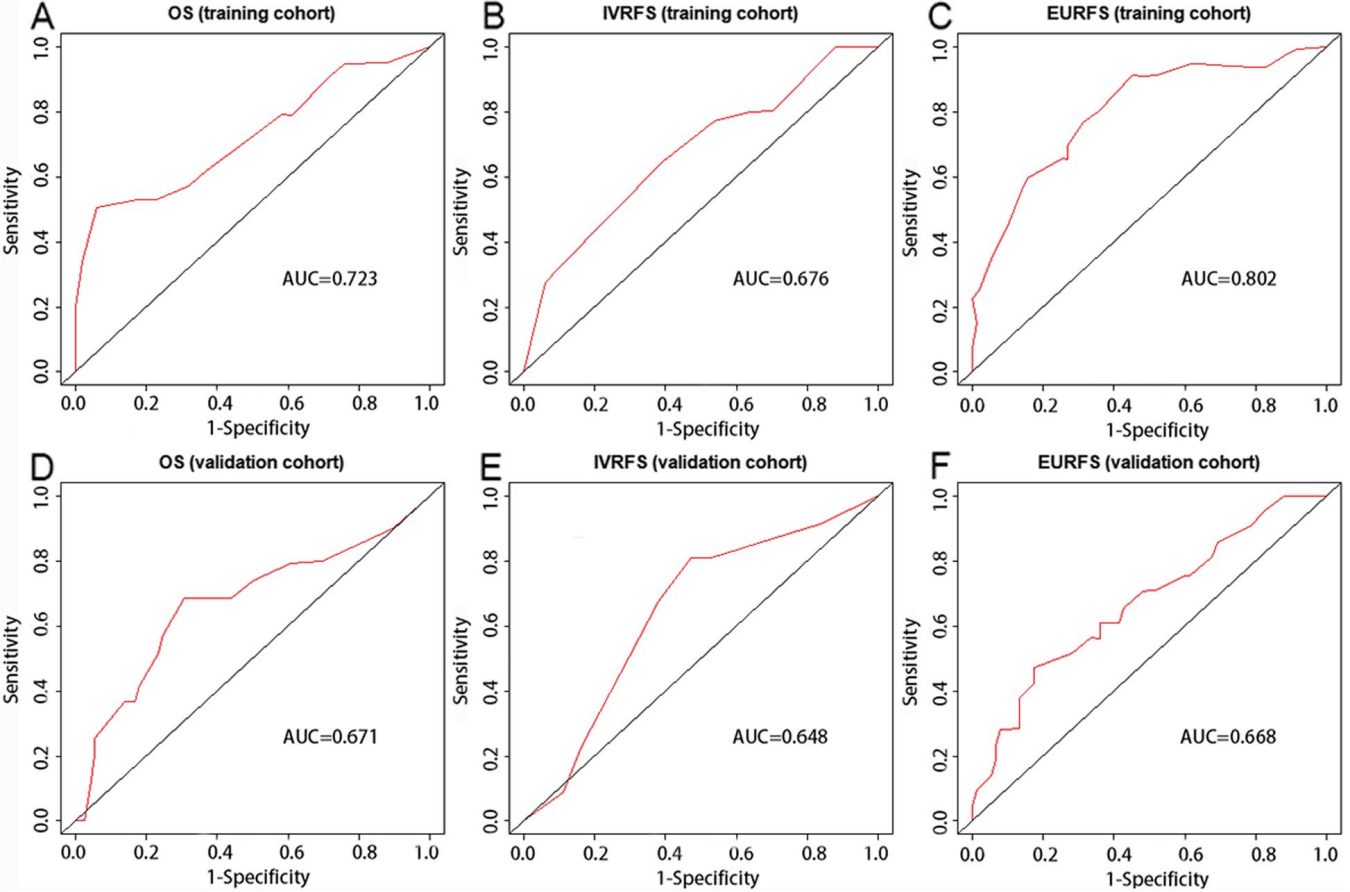
Nomogram ROC curves to predict OS in the training cohort (**A**) and validation cohort (**D**). Nomogram ROC curves to predict IVRFS in the training cohort (**B**) and validation cohort (**E**). Nomogram ROC curves to predict EURFS in the training cohort (**C**) and validation cohort (**F**)

The independent risk factors above were incorporated to construct the nomograms to predict OS, IVR, and EUR in patients with UTUC after RNU. The impact of each factor on the clinical outcomes was explicitly listed in the nomograms. The cumulative risk scores, obtained by summing individual risk scores, were subsequently calculated. Notably, the total risk points for predicting OS in the patients included in this study ranged from 0 to 293.4 (Fig. 1). Patients in the present study had total risk points for predicting IVR ranging from 0 to 191.3 (Fig. 2). Patients had total risk points for predicting EUR ranging from 0 to 343.9 (Fig. 3). The discriminative value of the nomogram was evaluated using the concordance index. In the training cohort, the C-index value for predicting OS was 0.675, while in the validation cohort, it was 0.715. For predicting IVR, the C-index value was 0.702 in the training cohort and 0.756 in the validation cohort. Similarly, for predicting EUR, the C-index value was 0.752 in the training cohort and 0.713 in the validation cohort. Model calibration was visually assessed through calibration curves (Fig. 4), which indicated satisfactory calibration of the new model. In the training set, the 3-year AUC values for the nomogram’s predictions of OS, IVR, and EUR were 0.723, 0.676, and 0.802, respectively. Similarly, in the validation set, the 3-year AUC values for the nomogram’s predictions of OS, IVR, and EUR were 0.671, 0.648, and 0.668, respectively (Fig. 5). These findings indicated that our nomograms exhibit favorable discriminatory ability.

### Clinical application of the nomograms

**Fig. 6:**
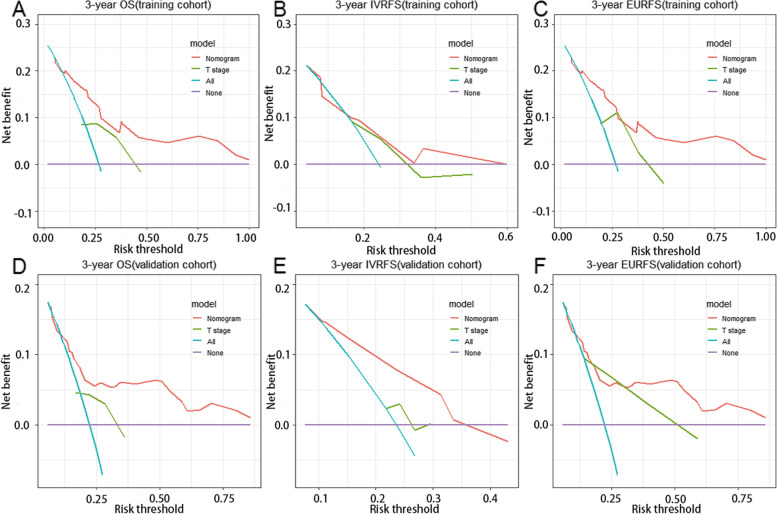
DCA analysis predicting 3-year OS in the training cohort (**A**) and validation cohort (**D**); DCA analysis predicting 3-year IVRFS in the training cohort (**B**) and validation cohort (**E**); DCA analysis predicting 3-year EURFS in the training cohort (**C**) and validation cohort (**F**)

We also estimated the IDI and NRI to compare the accuracy between the nomograms and the AJCC criteria-based pathological tumor staging alone. Using the nomogram in the training cohort, the NRI for the 3-year OS was 0.065, and the IDI value for the 3-year OS was 0.086. The NRI for the 3-year IVR was 0.296, and the IDI value for the 3-year IVR was 0.106. The NRI for the 3-year EURFS was 0.38, and the IDI value was 0.173. These results were validated in the validation cohort. NRI and IDI revealed improvements in discrimination (Table 3). The DCA of the training set and the validation set are shown in Fig. 6. When a threshold probability ranges from threshold 1 to threshold 2, using the nomogram to predict OS, IVRFS, and EURFS can achieve more benefits than using the pathological tumor stage alone. Finally, risk stratification was performed by calculating with the nomogram. In the training and validation cohorts, respectively, patients were divided into two risk groups: low-risk (total points ≤ 193.4, 100, and 138.3, for OS, IVRFS, and EURFS prediction, respectively) and high-risk group (total points > 193.4, 100, and 138.3). The Kaplan–Meier curves showed perfect discrimination among the two risk groups in both training and validation sets (Figure S2).

**Table 3:** NRI and IDI in training and validation cohort

	OS NRI	OS IDI	IVR NRI	IVR IDI	EUR NRI	EUR IDI
Training cohort	0.065	0.086	0.296	0.106	0.38	0.173
Validation cohort	0.076	0.031	0.357	0.071	0.193	0.099

*NRI* Net reclassification index, *IDI* Integrated discrimination improvement, *OS* Overall survival, *IVR* Intravesical recurrence, *EUR* Extraurothelial recurrence

### SII level and tumor invasion

The correlation between SII level and other clinical, pathological factors was shown in Table 4. Our analysis revealed a significant correlation between a high SII level and invasion in the mucosa, muscular layer of the ureter, fat tissues, and neural and vascular invasion. However, no correlation was observed between SII level and invasion in the subepithelial layer of the ureter, renal sinus, pararenal area, ureteral ends, and lymph node.

**Table 4:** Correlation of SII and other clinical, pathological factors

Variable	SII ≤ 470	SII > 470	*P* value
	*N* = 103	*N* = 92	
Gender			
Female	61 (59.2%)	50 (54.3%)	0.492
Male	42 (40.8%)	42 (45.7%)	
Age			
> 65	62 (60.2%)	53 (57.6%)	0.714
≤ 65	41 (39.8%)	39 (42.4%)	
Hypertension history			
No	52 (50.5%)	48 (52.2%)	0.814
Yes	51 (49.5%)	44 (47.8%)	
Diabetes history			
No	83 (80.6%)	75 (81.5%)	0.867
Yes	20 (19.4%)	17 (18.5%)	
BMI			
< 24	42 (40.8%)	46 (50.0%)	0.196
≥ 24	61 (59.2%)	46 (50.0%)	
Tumor side			
Left	57 (55.3%)	45 (48.9%)	0.370
Right	46 (44.7%)	47 (51.1%)	
Location			
Both	22 (21.4%)	18 (19.6%)	0.702
Renal pelvis	39 (37.9%)	31 (33.7%)	
Ureter	42 (40.8%)	43 (46.7%)	
Tumor size			
> 3	34 (33.0%)	37 (40.2%)	0.296
≤ 3	69 (67.0%)	55 (59.8%)	
Tumor stage			
≤ 2	77 (74.8%)	69 (75.0%)	0.969
≥ 3	26 (25.2%)	23 (25.0%)	
Neural or vascular invasion			
No	93 (90.3%)	73 (79.3%)	0.032
Yes	10 (9.71%)	19 (20.7%)	
Renal sinus invasion			
No	91 (88.3%)	83 (90.2%)	0.674
Yes	12 (11.7%)	9 (9.78%)	
Pararenal invasion			
No	91 (88.3%)	78 (84.8%)	0.464
Yes	12 (11.7%)	14 (15.2%)	
Mucosa invasion			
No	90 (87.4%)	69 (75.0%)	0.026
Yes	13 (12.6%)	23 (25.0%)	
Subepithelial invasion			
No	97 (94.2%)	90 (97.8%)	0.357
Yes	6 (5.83%)	2 (2.17%)	
Muscle invasion			
No	65 (63.1%)	44 (47.8%)	0.032
Yes	38 (36.9%)	48 (52.2%)	
Fat invasion			
No	95 (92.2%)	71 (77.2%)	0.003
Yes	8 (7.77%)	21 (22.8%)	
Cancer embolus			
No	94 (91.3%)	76 (82.6%)	0.071
Yes	9 (8.74%)	16 (17.4%)	
Ureteral ends invasion			
No	101 (98.1%)	87 (94.6%)	0.356
Yes	2 (1.94%)	5 (5.43%)	
Lymph node invasion			
No	101 (98.1%)	87 (94.6%)	0.356
Yes	2 (1.94%)	5 (5.43%)	
Hydronephrosis			
No	67 (65.0%)	57 (62.0%)	0.654
Yes	36 (35.0%)	35 (38.0%)	
Urine cytology			
Abnormal	54 (52.4%)	58 (63.0%)	0.134
Normal	49 (47.6%)	34 (37.0%)	
Ureteroscopy			
No	44 (42.7%)	38 (41.3%)	0.842
Yes	59 (57.3%)	54 (58.7%)	
ALT/AST			
> 0.55	67 (65.0%)	70 (76.1%)	0.092
≤ 0.55	36 (35.0%)	22 (23.9%)	
eGFR			
< 60	68 (66.0%)	62 (67.4%)	0.839
≥ 60	35 (34.0%)	30 (32.6%)	
Tumor size			
> 3	34 (33.0%)	37 (40.2%)	0.296
≤ 3	69 (67.0%)	55 (59.8%)	
Tumor grade			
High	87 (84.5%)	88 (95.7%)	0.020
Low	16 (15.5%)	4 (4.35%)	

## Discussion

Our study revealed that a high SII was a notable unfavorable prognostic determinant for OS and EUR in patients with UTUC after RNU. While certain factors such as tumor stage, tumor grade, and surgical margins have been associated with poor survival outcomes, these are typically assessed postoperatively using pathological specimens. In contrast, blood-based inflammation biomarkers can be conveniently obtained prior to surgery and aid urologists in making optimal clinical decisions for individual patients.

Inflammation and immune responses are critical components of tumor genesis, proliferation, invasion, and metastasis [22]. Inflammation-related indicators including SII, NLR, PLR, and LMR can reflect the situation of systemic inflammatory response and have been demonstrated to show prognostic value in in various malignancies [23–26]. The inflammation, infection, and oncogene activation lead to the activation of transcription factors in tumors and stroma, which subsequently lead to the production of chemokines, cytokines, and prostaglandins and induce the recruitment of inflammatory cells [27]. The secretion of chemokines and cytokines in the circulation mediates alteration in distant sites and results in tumor-derived cytokines and growth factors secreted into the systemic circulation to mediate alteration in distant sites [13]. Through the production of growth factors (for example G-CSF and GM-CSF) and the production of inflammatory cytokines, including IL-6, IL-1β, and IL-17 (neutrophil diversity and plasticity in tumor progression and therapy), tumor cells and tumor niche regulate the development, maturation, and release from the bone marrow of neutrophils, which result in peripheral neutrophilia [28, 29].

Neutrophils have complex roles in tumor development and progression. The pro-tumor phenotype of tumor-associated neutrophils can support tumor growth via different mechanisms, including the promotion of genetic instability, tumor cell proliferation, angiogenesis, metastasis, and immunosuppression [30, 31]. High infiltration of tumor-associated neutrophils and peripheral neutrophilia has been reported to be associated with poor prognosis in many human tumors [32, 33]. It has also been reported that high NLR and increased peripheral blood neutrophil counts may be associated with a higher frequency of tumor-infiltrating neutrophils [34]. Peripheral neutrophils also contribute to tumor development, progression, and metastasis through a variety of mechanisms, including the promotion of angiogenesis, production of matrix metalloproteinases, and escorting of circulating tumor cells [35–37].

Platelets play an important role in tumor progression. Paracrine secretion of IL-6 from tumor cells stimulates the production of thrombopoietin (TPO), resulting in megakaryopoiesis and platelet genesis and leading to a status of thrombocytosis and hypercoagulability known as Trousseau’s syndrome [38, 39]. Platelets can directly or indirectly interact with tumor cells and increase tumor progression by promoting proliferation, resisting cell death, inducing angiogenesis, activating invasion, establishing pre-metastatic microhabitats, and evading immune detection [40]. Elevated platelet counts have been reported to be associated with increased cancer risk at several sites [41].

Numerous studies have reported associations between elevated platelet counts and decreased disease-specific survival rates across various types of cancer [42]. In the context of cancer immune surveillance and resistance, lymphocytes play a crucial role in impeding the proliferation and growth of tumor cells through cytotoxic cell death. Conversely, the presence of T lymphocytes within the tumor microenvironment has been consistently linked to improved prognoses, highlighting their significant anti-tumor functionality [43, 44]. Lymphocytes inhibit the proliferation and growth of tumor cells by cytotoxic cell death in cancer immune surveillance and resistance. In contrast, lymphocytes have an important anti-tumor function, and infiltration of T lymphocytes in the tumor microenvironment was known to be correlated with better prognosis [43, 44]. CD8 + T cells contribute to direct tumor cell lysis and the production of cytotoxic cytokines. CD4 + Th1 cells assist cytotoxic T lymphocytes and impress tumor progression by the production of cytokines (for example, IFN-γ), Th17 cells, and Treg cells function in the anti-tumor process by activating cytotoxic lymphocytes or suppression of inflammation [45]. To summarize, neutrophils, platelets, and lymphocytes are crucial components in inflammation and immunity related to cancer. In UTUC, multiple system inflammation and immune-related indexes based on these factors have been developed to predict the prognosis of patients after RNU, including NLR, PLR, and LMR[7, 46–48]. The SII, which incorporates the counts of neutrophils, platelets, and lymphocytes, offers a more comprehensive assessment of the host’s immune and inflammatory status compared to the aforementioned indicators [49].

For patients with UTUC after RNU, IVR and EUR can significantly decrease survival time. Therefore, the relative prediction model also aroused a great interest in recent years. To our knowledge, the present study is the first proposal to confirm that SII possesses predictive value for EUR among UTUC patients and construct a prediction model with SII included. In accordance with prior research, our findings indicated that urine cytology, eGFR, and tumor stage were independent prognostic factors for IVR [48]. Interestingly, SII was not associated with IVR from our results. Although Chen et al. reported elevated SII can predict bladder recurrence, some patients with a history of bladder cancer were included in their study [11]. We hypothesize that the observed disparity may be attributed to the heterogeneity of the study population, variations in baseline characteristics, inclusion of different variables, inadequate sample size, or statistical noise. Undoubtedly, further investigations involving larger sample sizes are imperative to establish reliable conclusions.

In addition, an examination was conducted to investigate the correlation between preoperative SII levels and invasion sites. The outcomes revealed a significant association between elevated SII levels and high tumor grade, as well as invasion in various anatomical locations including the mucosa, muscle, adipose tissue, and neural and vascular structures. These findings suggested a heightened invasiveness of tumors exhibiting elevated SII levels. Plausible mechanistic explanations for these observations involve tumor-induced inflammation and subsequent cytokine production, particularly IL-6 and IL-8, which are known to play a pivotal role in the epithelial-mesenchymal transition (EMT). Furthermore, the induction and maintenance of tumor EMT are facilitated by the presence of inflammation, thereby facilitating the advancement towards metastasis [50].

There are several limitations to the present study that should be acknowledged. Firstly, it is imperative to note that this study is retrospective and conducted within a single center, thus potentially limiting the generalizability of the findings due to the relatively small sample size. Secondly, the inclusion of only 11 patients with low-grade tumor grade necessitates further validation of the predictive capabilities of the models for clinical outcomes in patients with low tumor grade. Lastly, it is crucial to develop a more universally applicable threshold for SII, as the cutoff employed in this study may not be applicable across other studies.

## Conclusion

Our study suggested that a high level of preoperative SII is associated with both worse OS and shorter EURFS in UTUC patients after RNU. We developed nomogram models for predicting the OS, IVR, and EUR of patients, respectively, and their discrimination, calibration, and clinical use were proved through internal validation.

### Supplementary Information

**Additional file 1: Figure S1. **LASSO coefficient profiles of all variables predicting OS (A), 10-fold cross-validation for tuning parameter selection in the least LASSO model related to OS (B); LASSO coefficient profiles of the variables predicting IVRFS (C), 10-fold cross validation for tuning parameter selection in the least LASSO model related to IVRFS (D); LASSO coefficient profiles of the variables predicting EURFS (E), 10-fold cross validation for tuning parameter selection in the least LASSO model related to EURFS (F).

**Additional file 2: Figure S2. **The Kaplan-Meier curves of OS nomogram in the training cohort (A) and validation cohort (D); The Kaplan-Meier curves of IVRFS nomogram in the training cohort (B) and validation cohort (E); The Kaplan-Meier curves of EURFS nomogram in the training cohort (C) and validation cohort (F).

**Additional file 3: Table S1. **Univariate and multivariate analyses of predictive factors for intravesical recurrence.

**Additional file 4: Table S2.** Univariate and multivariate analyses of predictive factors for extraurothelial recurrence.

## Data Availability

The datasets used and/or analyzed during the current study are available from the corresponding author on reasonable request.

## Abbreviations

EUR: Extraurothelial recurrence
UTUC: Upper tract urothelial carcinoma
RUN: Radical nephroureterectomy
EAU: European Association of Urology
IVR: Intravesical recurrence
CT: Computed tomography
NLR: Neutrophil-to-lymphocyte ratio
PLR: Platelet-to-lymphocyte ratio
LMR: Lymphocyte-to-monocyte ratio
SII: Systemic immune-inflammation index
RFS: Recurrence-free survival
CSS: Cancer-specific survival
OS: Overall survival
EUR: Extraurothelial recurrence
IVRFS: Intravesical recurrence-free survival
EURFS: Extraurothelial recurrence-free survival
ALT/AST: Aspartate transaminase/alanine transaminase
DM: Diabetes mellitus
BMI: Body mass index
eGFR: Estimated glomerular filtration rate
ROC: Receiver operating characteristic
AJCC: American Joint Committee on Cancer
TNM: Tumor, node, metastasis
WHO: World Health Organization
LASSO: Least absolute shrinkage and selection operator
VIF: Variance inflation factor
C-index: Concordance indexes
AUC: Area under the curve
IQRs: Interquartile ranges
NRI: Net reclassification index
IDI: Integrated discrimination improvement
DCA: Decision curve analysis
TPO: Thrombopoietin
EMT: Epithelial-mesenchymal transition
