# Supplementary figures and images for: Prognostic value of the systemic immune-inflammation index in patients with upper tract urothelial carcinoma after radical nephroureterectomy
Source: World J Surg Oncol. 2023 Oct 26;21:337. doi: 10.1186/s12957-023-03225-0 (PMC10601258; doi:10.1186/s12957-023-03225-0)

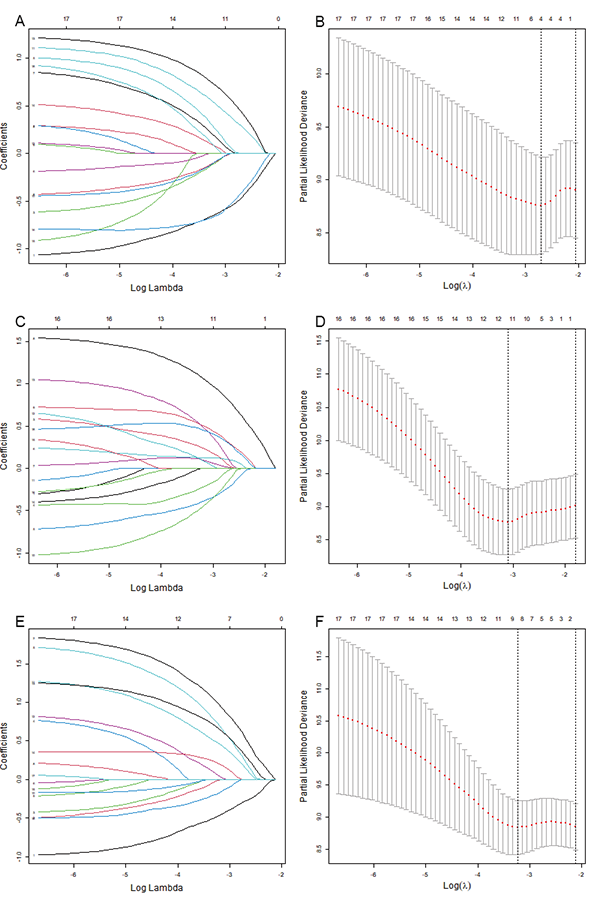

Supplement: Supplementary file 1 — Additional file 1: Figure S1. LASSO coefficient profiles of all variables predicting OS (A), 10-fold cross-validation for tuning parameter selection in the least LASSO model related to OS (B); LASSO coefficient profiles of the variables predicting IVRFS (C), 10-fold cross validation for tuning parameter selection in the least LASSO model related to IVRFS (D); LASSO coefficient profiles of the variables predicting EURFS (E), 10-fold cross validation for tuning parameter selection in the least LASSO model related to EURFS (F). [file 12957_2023_3225_MOESM1_ESM.tif]

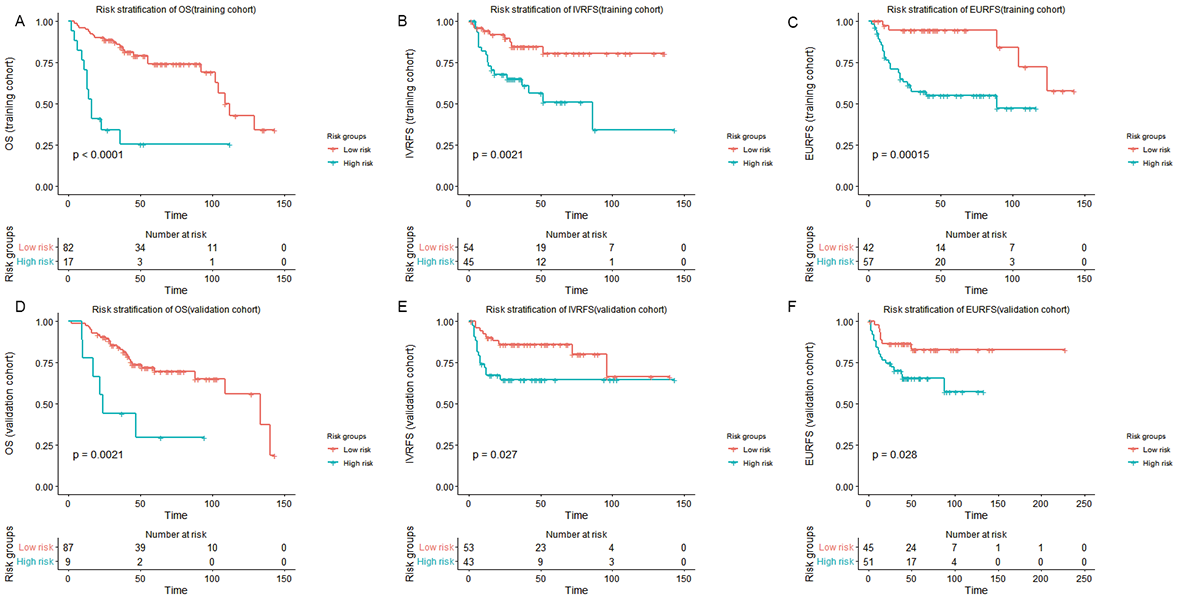

Supplement: Supplementary file 2 — Additional file 2: Figure S2. The Kaplan-Meier curves of OS nomogram in the training cohort (A) and validation cohort (D); The Kaplan-Meier curves of IVRFS nomogram in the training cohort (B) and validation cohort (E); The Kaplan-Meier curves of EURFS nomogram in the training cohort (C) and validation cohort (F). [file 12957_2023_3225_MOESM2_ESM.tif]
