# Supplementary material for: Prognostic value of the systemic immune-inflammation index in patients with upper tract urothelial carcinoma after radical nephroureterectomy
Source: World J Surg Oncol. 2023 Oct 26;21:337. doi: 10.1186/s12957-023-03225-0 (PMC10601258; doi:10.1186/s12957-023-03225-0)
Supplement: Supplementary file 3 — Additional file 3: Table S1. Univariate and multivariate analyses of predictive factors for intravesical recurrence. [file 12957_2023_3225_MOESM3_ESM.docx]

| **Table S1 Univariate and multivariate analyses of predictive factors for intravesical recurrence** | | | | |
| --- | --- | --- | --- | --- |
| Variable | P value | HR (95% CI for HR) | P value | HR (95% CI for HR) |
| Age | 0.62 | 0.82 (0.37-1.8) |  |  |
| BMI | 0.092 | 0.52 (0.24-1.1) | 0.19 | 0.53 (0.2-1.4) |
| Urine cytology | 0.0042 | 0.21 (0.072-0.61) | 0.0051 | 0.2 (0.067-0.62) |
| Diabetes history | 0.17 | 0.43 (0.13-1.4) | 0.4 | 0.56 (0.14-2.2) |
| eGFR | 0.16 | 0.52 (0.21-1.3) | 0.048 | 0.35 (0.13-0.99) |
| Gender | 0.83 | 0.92 (0.43-2) |  |  |
| Tumor grade | 0.38 | 0.41 (0.055-3) |  |  |
| Hypertension history | 0.13 | 1.8 (0.84-3.9) | 0.24 | 1.7 (0.7-4) |
| ALT/AST | 0.37 | 0.66 (0.27-1.6) | 0.36 | 0.64 (0.25-1.7) |
| Hydronephrosis | 0.1 | 1.9 (0.88-4.1) | 0.83 | 1.1 (0.4-3.1) |
| Renal pelvic carcinoma | 0.15 | 0.53 (0.22-1.3) |  |  |
| Ureteral carcinoma | 0.67 | 0.84 (0.38-1.9) |  |  |
| Tumor in both | 0.032 | 2.3 (1.1-5) | 0.17 | 1.9 (0.76-4.6) |
| Tumor side | 0.065 | 2.1 (0.95-4.6) | 0.73 | 1.2 (0.47-2.9) |
| SII | 0.5 | 0.77 (0.36-1.6) |  |  |
| Tumor size | 0.39 | 1.5 (0.6-3.7) | 0.49 | 1.4 (0.52-3.9) |
| Tumor stage | 0.22 | 1.6 (0.74-3.7) | 0.019 | 3 (1.2-7.3) |
| Ureteroscopy | 0.036 | 2.6 (1.1-6.6) | 0.095 | 2.3 (0.86-6.3) |

BMI, body mass index; SII, Systemic immune-inflammation index; ALT/AST, serum aspartate transaminase/alanine transaminase; eGFR, estimated glomerular filtration rate
