# Supplementary material for: Prognostic value of the systemic immune-inflammation index in patients with upper tract urothelial carcinoma after radical nephroureterectomy
Source: World J Surg Oncol. 2023 Oct 26;21:337. doi: 10.1186/s12957-023-03225-0 (PMC10601258; doi:10.1186/s12957-023-03225-0)
Supplement: Supplementary file 4 — Additional file 4: Table S2. Univariate and multivariate analyses of predictive factors for extraurothelial recurrence. [file 12957_2023_3225_MOESM4_ESM.docx]

| **Table S2 Univariate and multivariate analyses of predictive factors for extraurothelial recurrence** | | | | |
| --- | --- | --- | --- | --- |
| Variable | P value | HR (95% CI for HR) | P value | HR (95% CI for HR) |
| Age | 0.63 | 1.2 (0.58-2.4) |  |  |
| BMI | 0.18 | 1.7 (0.78-3.6) | 0.0023 | 4.8 (1.8-13) |
| Urine cytology | 0.84 | 0.93 (0.44-1.9) |  |  |
| Diabetes history | 0.7 | 0.82 (0.31-2.2) |  |  |
| eGFR | 0.57 | 0.8 (0.36-1.7) |  |  |
| Gender | 0.044 | 2.1 (1-4.4) | 0.044 | 2.3 (1-5.1) |
| Tumor grade | 0.4 | 0.42 (0.057-3.1) |  |  |
| Hypertension history | 0.56 | 0.8 (0.39-1.7) |  |  |
| ALT/AST | 0.72 | 0.86 (0.36-2) | 0.27 | 0.59 (0.23-1.5) |
| Hydronephrosis | 0.029 | 2.2 (1.1-4.6) | 0.0015 | 5.8 (2-17) |
| Renal pelvic carcinoma | 0.25 | 0.63 (0.29-1.4) | 0.32 | 0.62 (0.25-1.6) |
| Ureteral carcinoma | 0.18 | 1.6 (0.8-3.4) |  |  |
| Tumor in both | 0.85 | 0.92 (0.4-2.2) |  |  |
| Tumor side | 0.79 | 1.1 (0.54-2.3) |  |  |
| SII | 0.17 | 0.6 (0.29-1.3) | 0.028 | 0.39 (0.17-0.9) |
| Tumor size | 0.36 | 0.71 (0.34-1.5) | 0.15 | 0.57 (0.26-1.2) |
| Tumor stage | 0.085 | 1.9 (0.91-4.1) | 0.0069 | 3 (1.4-6.8) |
| Ureteroscopy | 0.53 | 1.3 (0.6-2.7) |  |  |

BMI, body mass index; SII, Systemic immune-inflammation index; ALT/AST, serum aspartate transaminase/alanine transaminase; eGFR, estimated glomerular filtration rate
